# Egg size and the adaptive capacity of early life history traits in Chinook salmon (*Oncorhynchus tshawytscha*)

**DOI:** 10.1111/eva.12531

**Published:** 2017-09-14

**Authors:** Michael W. Thorn, Yolanda E. Morbey

**Affiliations:** ^1^ Department of Biology University of Western Ontario London ON Canada

**Keywords:** divergence, early life history, egg size, evolution, heritability, maternal effect, quantitative genetics, salmon

## Abstract

Offspring traits are greatly influenced by maternal effects, and these maternal effects may provide an important pathway through which populations can adapt to changing thermal environments. We investigated the effect of egg size on the among‐ and within‐population variation in early life history traits among introduced Great Lakes Chinook salmon (*Oncorhynchus tshawytscha*) populations under varying thermal conditions. We reared Chinook salmon from three populations in a common‐garden hatchery study at 6.5, 9.4, and 15.2°C and measured a variety of fitness‐related traits during development. We found that most of the among‐population variation in early life history traits was explained by egg size. However, the contribution of egg size to the among‐population variation decreased with an increase in temperature suggesting that other effects, such as genetic, contribute at high temperature. Within populations, egg size explained much of the dam variance and maternal effect for traits in every temperature, whereas egg size generally had little to no influence on the sire variance and heritability. Overall, our results demonstrate the significant contribution egg size makes to shaping early life history phenotypes among and within populations, and suggest that egg size is an important pathway through which offspring phenotypes can evolve on contemporary timescales.

## 1 INTRODUCTION

Temperature has a direct effect on the rate of biological reactions within the body of ectotherms (Gillooly, Brown, West, Savage, & Charnov, 2001; Huey & Kingsolver, 1989), and alterations to environmental temperature regimes, such as those predicted for climate change, can have a dramatic impact on the development, fitness, and lifespan of these organisms (Andrews & Schwarzkopf, 2012; Clusella‐Trullas, Blackburn, & Chown, 2011; Fry, 1967; Munch & Salinas, 2009; Paaijmans et al., 2013; Wood & McDonald, 1997). Temperature variation does not necessarily affect all life stages equally and the early life history stages are often the most vulnerable because offspring tend to have a low tolerance for temperature variation (Rombough, 1997; Xu & Ji, 2006) and a reduced capacity to behaviorally thermoregulate (Quinn, 2005). Furthermore, offspring tend to experience strong selection pressures, such as size‐selective mortality (Elliott, 1990; Sogard, 1997), and a temperature‐mediated shift in offspring phenotype or habitat can reduce offspring fitness (Crozier et al., 2008; Ficke, Myrick, & Hansen, 2007; Massot, Clobert, & Ferrière, 2008; Visser & Both, 2005). A reduction in survival during early life can have a strong negative effect on recruitment and threaten the persistence of a population (Kareiva, Marvier, & McClure, 2000; Venturelli et al., 2010; Zabel, Scheuerell, McClure, & Williams, 2006). Populations facing such a predicament will need to adaptively respond by shifting offspring phenotypes on ecological timescales. To understand the adaptive capacity of offspring traits, we need to know the sources of phenotypic variation underlying the traits and how the contribution of these sources is influenced by temperature.

In fishes, egg size has been shown to affect the variation in offspring phenotypes, whereby there is positive relationship between egg size and offspring size (Chambers & Leggett, 1996; Heath & Blouw, 1998). Large juveniles often experience increased competitive ability (Cutts, Brembs, Metcalfe, & Taylor, 1999), growth (Einum & Fleming, 1999), swimming performance (Ojanguren, Reyes‐Gavilán, & Braña, 1996), and survival (Sogard, 1997). However, any developmental and fitness advantages of large egg size are context‐dependent and can vary across environmental gradients (temperature: Beacham & Murray, 1985; Régnier, Bolliet, Gaudin, & Labonne, 2013; habitat: Einum & Fleming, 1999; food resources: Segers & Taborsky, 2011). For example, Beacham and Murray (1985) found that the size of chum salmon (*Oncorhynchus keta*) juveniles was positively related to egg size at 4 and 8°C, whereas there was no relationship at 12°C (i.e., large and small eggs produce similar‐sized juveniles). The phenotypic variation explained by egg size has often been quantified using a regression between egg size and an offspring trait, which provides an estimation of the total variation explained by egg size (i.e., *R* ^2^; Heath & Blouw, 1998) and does not partition the variation explained among and within populations. Furthermore, few studies have controlled for the breeding design employed, which may result in the inaccurate estimation of the egg size effect (Burt, Hinch, & Patterson, 2011). Given the influence egg size has on offspring phenotypes, quantifying the contribution of egg size to the among‐ and within‐population variation is crucial for our understanding of early life history trait evolution.

Quantitative genetic studies provide information on the genetic, maternal, and environmental sources of phenotypic variation within a population, which is necessary to elucidate the potential pathways through which populations may respond to a change in temperature (Lynch & Walsh, 1998). Quantitative genetic studies of early life history traits have shown that these traits are largely influenced by maternal rather than genetic effects (Falica, Lehnert, Pitcher, Heath, & Higgs, 2016; Heath, Fox, & Heath, 1999; Houde, Wilson, & Neff, 2013; Kinnison, Unwin, Hershberger, & Quinn, 1998; Páez, Morrissey, Bernatchez, & Dodson, 2010; Pitcher & Neff, 2007). Often, these studies quantify the maternal effect as the proportion of phenotypic variation explained by dam identity, which does not provide information on the maternal effect traits that contribute to the overall maternal effect (McAdam, Garant, & Wilson, 2014). Haugen and Vøllestad (2000) found that egg size does make a significant contribution to the maternal variation in graying (*Thymallus thymallus*) early life history traits, but were unable to quantify the amount of maternal variation explained by egg size. Another limitation of many quantitative genetic studies is the use of a single thermal environment (Burt et al., 2011). Natural environments are rarely static, and the rearing environment can greatly influence the estimation of quantitative genetic parameters (Carlson & Seamons, 2008; Charmantier & Garant, 2005; Hoffmann & Merilä, 1999). As a result, the contribution of egg size to the within‐population variation in offspring traits may vary across a thermal gradient.

Quantifying the among‐population variation in offspring traits explained by egg size can provide information about the capacity of egg size to alter these traits. Frequently, studies of contemporary evolution are specifically interested in genetic differences among populations and merely control for egg size effects when comparing traits among populations (Hendry, Hensleigh, & Reisenbichler, 1998; Jensen et al., 2008; Kinnison et al., 1998). Those that are interested in egg size often use statistical inference or qualitative assessments to determine how egg size contributes to the among‐population variation in offspring traits (Ghani, Izza, Herczeg, & Merilä, 2012; Jones & Closs, 2016; Koskinen, Haugen, & Primmer, 2002). Using a different approach, Aykanat, Bryden, and Heath (2012) crossed males and females from several Chinook salmon populations (*Oncorhynchus tshawytscha*) in a factorial breeding design and found that among‐population maternal effects, but not specifically egg size, explained most of the observed population differences in offspring traits. Although egg size has commonly been implicated in the among‐population variation in offspring traits, a measure of effect size is generally lacking and is required to fully understand how egg size can contribute to population divergence.

In the Laurentian Great Lakes, Chinook salmon populations were first introduced in the late 1960s from the Green River, Washington (Parsons, 1973; Weeder, Marshall, & Epifanio, 2005). This introduction represents one of the largest ecosystem manipulations in the world. Since their introduction, Chinook salmon have colonized tributaries throughout the Great Lakes, and there is now evidence of high natural reproduction (Connerton, Murry, Ringler, & Stewart, 2009; Johnson, DeWitt, & Gonder, 2010). There is also evidence of weak genetic structuring among the populations (Suk, Neff, Quach, & Morbey, 2012), suggesting that there is potential for phenotypic divergence of early life history traits. For this study, we used introduced Great Lakes Chinook salmon populations to test several hypotheses: (i) the divergence in early life history traits among introduced Chinook salmon populations will be largely mediated by variation in egg size; (ii) egg size will also influence the estimation of quantitative genetic parameters (i.e., within‐population variation); and (iii) the variance explained by egg size, both among and within populations, will depend on the rearing temperature of the offspring. We reared progeny from three Great Lakes Chinook salmon populations in a common‐garden hatchery experiment under three different temperature regimes and measured a variety of fitness‐related early life history traits. We then used a model comparison approach, whereby we compared models before and after including egg size, to quantify the variation among and within populations that is explained by egg size across the three temperature treatments.

## 2 MATERIALS AND METHODS

### 2.1 Study populations

**Figure 1:**
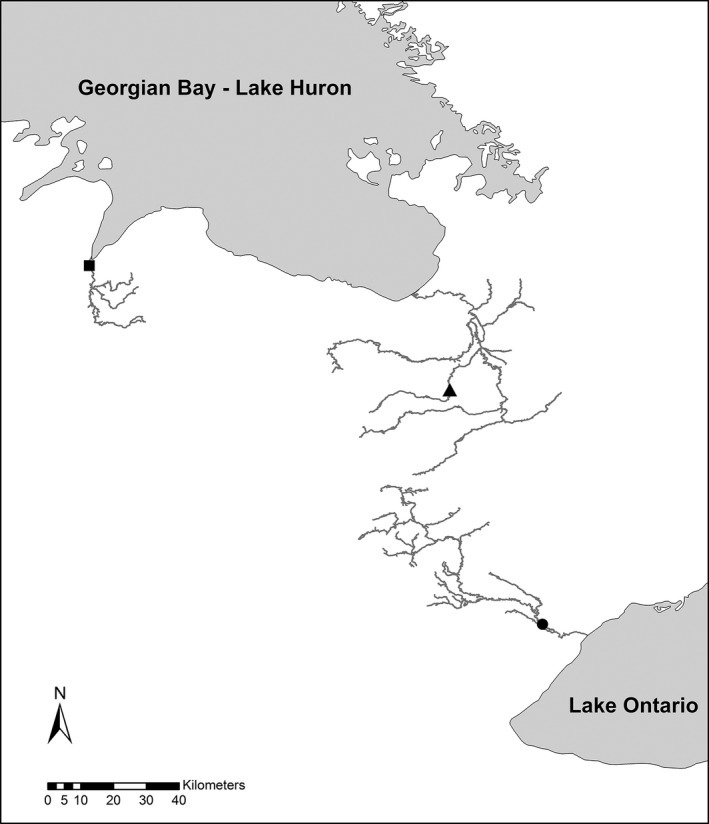
Location of the Credit River (circle), Pine River (triangle), and Sydenham River (square) Chinook salmon collection sites. All sites were located in Ontario, Canada. The map was created using publicly available data in ArcMap 10.3 (ESRI, 2015) and projected using UTM NAD83 zone 17N. River systems were simplified for display purposes

The populations used in this study were from the Credit River (CR), Pine River (PR), and Sydenham River (SR; Figure 1). Chinook salmon were introduced to Lake Huron by the Michigan Department of Natural Resources starting in 1968 using embryos from the Green River, Washington (Parsons, 1973; Weeder et al., 2005). Stray Chinook salmon from Michigan stocking operations eventually colonized the PR and SR in southern Georgian Bay, Lake Huron, in ~1980 (Kerr & Perron, 1986; Suk et al., 2012). Chinook salmon were then introduced to Lake Ontario in 1969 via the Little Salmon River, New York, using a combination of Chinook salmon from established populations in Michigan (probably from Lake Huron) and embryos sent from the Green River, Washington (Donaldson & Timothy, 1983). In 1982, the Ontario Ministry of Natural Resources and Forestry initiated a Chinook salmon stocking program in the CR using previously established Lake Ontario Chinook salmon populations (Daniels & LeTendre, 1987; FWS/GLFC, 2010). Therefore, all the populations used in this study descended from the Green River, Washington population. The populations have been separated for ~30 years, which translates into ~10 generations using an estimated generation time of 3 years (Haring, Johnston, Wiegand, Fisk, & Pitcher, 2016; Suk et al., 2012).

The migration distance and timing differ among the study populations, whereas the rivers have similar thermal profiles. The SR has the shortest migration distance of ~7 kilometers (km), followed by the CR at ~14 km, and the PR has the longest migration of >100 km. The PR population arrives at their spawning grounds as early as mid‐August, whereas the CR and SR typically arrive in late September (Gerson, Marklevitz, & Morbey, 2016; M. Thorn, personal observation). Thermal profiles are similar among the rivers, with mean (±*SE*) water temperature between mid‐October 2010 and May 2011 of 2.9°C ± 0.30, 2.5°C ± 0.21, and 3.1°C ± 0.23 for the CR, PR, and SR, respectively (Fig. S1).

### 2.2 Gamete collection

Chinook salmon were collected at the Streetsville Dam in the CR using electrofishing (43°34′39.58″N, 79°42′8.57″W), at the Mill Street Dam in the SR using a fish trap built into the dam (44°33′34.36″N, 80°56′39.49″W), and at the PR using a combination of dip and seine nets (44°13′10.12″N, 79°57′24.84″W). Because of differences among the populations in run timing, adults were collected in the CR on October 01, 2012, in the PR from September 19 to 27, 2012, and in the SR from September 22 to October 06, 2012. When an adult was captured, it was anesthetized by immersing it in a clove oil solution (20 mg/L), measured for fork length and mass, and checked for sexual maturity. If the individual was found to be sexually mature, a sample of eggs or sperm was collected by gently massaging the abdomen. Approximately 500 eggs and a few milliliters of milt were taken from each female and male, respectively. Visually unhealthy salmon were not used. All collected fish were released once gamete sampling was complete. Egg and milt samples were stored in a cooler (~4°C) and transported directly to the Western University experimental hatchery for fertilization within 8 hr of collection.

### 2.3 Hatchery experiment

Eggs from each population were partitioned into separate egg containers (40 eggs per container; 6 cm diameter × 5 cm height) according to maternal origin (six containers per female) and then fertilized using a nested full‐sib, half‐sib breeding design (one male × two females; Lynch & Walsh, 1998). The fertilization procedure yielded 20 CR (10 males × 20 females), 26 PR (13 males × 26 females), and 22 SR families (11 males × 22 females). Two egg containers from each family (80 fertilized eggs) were placed in upwelling incubation trays at a mean temperature (°C ± *SD*) of 6.5 ± 0.8, 9.4 ± 0.3, and 15.2 ± 0.02 (i.e., two containers from each female at each temperature). These temperatures were chosen because they represent the range of temperatures the three populations experience in the wild during incubation (Fig. S1). The 15.2°C treatment reflects the warm water temperatures experienced by embryos early during incubation and later into the summer as free‐feeding juveniles (~June). The 9.4°C treatment is a mid‐range temperature that is close to the optimum growth temperature of Chinook salmon (Richter & Kolmes, 2005). The 6.5°C treatment was the lowest possible temperature we could achieve in the hatchery and reflects the lower range of temperatures these populations can experience during incubation. A sample of 25 eggs from each female was also retained and measured for egg diameter using handheld calipers to the nearest 0.1 mm. The developing embryos were checked daily, and all dead/unfertilized eggs were removed. The removed eggs were stored in Stockard's solution and later checked for evidence of embryonic development (Boyd, Oldenburg, & McMichael, 2010). This allowed us to calculate fertilization success and embryo mortality for each egg container. After hatch, the developing alevins remained in the egg containers until they had reached the swim‐up stage, which is when the fish have absorbed the yolk sac, are neutrally buoyant, and begun free feeding. The fish were then transferred family‐wise to larger containers (10.5 cm diameter × 35 cm height) suspended in large recirculating tanks set at the same thermal regimes as the vertical incubators the families originated from. At this time, one egg container per family at each temperature was chosen at random and the individuals euthanized for swim‐up measurements. The transferred fish were fed ad libitum in the large containers until the termination of the experiment at 300 degree‐days posthatch, where the remaining individuals were euthanized for juvenile measurements. No fish in the warm treatment were sampled at the juvenile stage because of a large die off that occurred prior to the termination of the experiment. All procedures in this study were approved by the Western University Animal Use Subcommittee.

### 2.4 Trait measurements

A variety of fitness‐related early life history traits were measured during the experiment: hatch length, yolk sac volume, yolk sac conversion efficiency, swim‐up length, hatch to swim‐up growth rate, juvenile length, and swim‐up to juvenile growth rate. All length measurements were taken from the anterior tip of the snout to the posterior tip of the hypural plate (i.e., standard length). Hatch length and yolk sac volume were measured from digital photographs taken of each family next to a ruler in a petri dish with water using the computer program ImageJ (http://imagej.nih.gov/ij/). Yolk sac volume was estimated as:

$$V\;=\;\left(\pi /6\right)\;\times \;L\;\times \;H^{2}$$

where *L* is the yolk sac length (mm) and *H* is the yolk sac height (mm; Blaxter & Hempel, 1963). Yolk sac conversion efficiency was estimated as:

$$Y\;=\;(L_{S}-L_{H})/V$$

where *L*_S_ is swim‐up length (mm), *L*_H_ is hatch length (mm), and *V* is the yolk sac volume (mm^3^; Fraser et al., 2010). Swim‐up and juvenile length were measured using handheld calipers to the nearest 0.1 mm. Growth rates were calculated as:

$$G\;=\;(L_{2}-L_{1})/\Delta D$$

where *L*_2_ is the length of the later life history stage (mm), *L*_1_ is the length of the earlier life history stage (mm), and ∆*D* is the growing degree‐days between the two life history stages (Jensen et al., 2008). The growing degree‐days were measured as the cumulative sum of mean daily temperature up to a given time period (Jensen et al., 2008). Wet mass was measured using a Mettler‐Toledo AL204 analytical balance to the nearest 0.001 g. The number of offspring measured for a given trait varied per family and population depending on mortality at each temperature and stage of development. The sample size information for each trait/population/temperature combination is provided in the supplementary materials (Table S1).

### 2.5 Egg size comparison among populations

All analyses were conducted using the R statistical computing environment (version 3.2.5; R Core Team, 2016). Egg diameter was compared among populations using one‐way analysis of variance (ANOVA) and an analysis of covariance (ANCOVA) with female fork length as a covariate followed by post hoc Tukey tests. Female length was included in the analysis because egg size has been found to be positively related to female body size (van den Berghe & Gross, 1989). The fork length × population interaction was found to be nonsignificant (*p* > .05) and was dropped from the analysis.

### 2.6 Egg size and multivariate trait comparisons

To assess the differences in early life history traits among populations, we used nonmetric multidimensional scaling (nMDS) ordination based on a Euclidean distance matrix and a permutational multivariate analysis of variance (PERMANOVA) using the vegan package in R (Oksanen et al., 2016). We used a multivariate approach to evaluate population differences because the analysis incorporates all the traits and provides a more holistic picture relative to comparing individual traits. nMDS was selected instead of an eigenvector‐based method, such as principal components analysis, because we were interested in using all the variance in the early life history data to visualize the distance among populations and not just part of the variance associated with a subset of gradients (Paliy & Shankar, 2016). A Euclidean distance matrix was created using all the morphological and growth‐related traits measured at a given temperature treatment. The trait data were standardized into *z*‐scores prior to calculating the Euclidean distance matrix. An nMDS ordination was considered acceptable if the stress value was ≤15 (Clarke, 1993). A primary assumption of the PERMANOVA test is the homogeneity of dispersion, and we tested this assumption using a multivariate version of Levene's test (vegan function; Oksanen et al., 2016). We found that none of the trait matrices violated this assumption (*p* > .05). The PERMANOVA was first run on the full early life history trait matrix using population as a fixed effect to see whether there was an overall effect of population. If the population effect was significant, we repeated the PERMANOVA on all pairwise population combinations to determine which populations were different. The analysis was run for each temperature separately.

We then used a Mantel test to determine whether there was a correlation between the early life history trait distance matrix and an egg size distance matrix (both Euclidean distance matrices). The significance of the Mantel test was determined by permuting one of the distance matrices 999 times (vegan function; Oksanen et al., 2016). We ran a separate Mantel test for each temperature treatment.

### 2.7 Egg size and among‐population variation

We used a model comparison approach to evaluate the effects of egg size on the among‐population variation in early life history traits. We did this by comparing models before and after controlling for egg size using linear mixed models. Length and volume trait data were collected at the individual level, whereas the growth traits were derived measures at the family level. As a result, we had to specify different models for the individual‐ and family‐level traits. The linear mixed models used to compare the individual‐level traits before (1) and after (2) controlling for egg size were as follows:

(1) $$Z_{ijklm}\;=\;\mu \;+\;P_{i}\;+\;V_{S_{ij}}\;+\;V_{D_{ijk}}\;+\;V_{c_{ijkl}}\;+\;V_{E_{ijklm}}$$

(2) $$Z_{ijklm}\;=\;\mu \;+\;P_{i}\;+\;\text{EG}\;+\;P\;\times \;\text{EG}\;+\;V_{S_{ij}}\;+\;V_{D_{ijk}}\;+\;V_{C_{ijkl}}\;+\;V_{E_{ijklm}}$$

where *z*_*ijklm*_ is the phenotype of the *m*th offspring of the *i*th population, *j*th sire, *k*th dam, and *l*th container, *P* is the fixed effect of population, EG is the egg size covariate, *P *× EG is the interaction between population and egg size, $V_{S_{ij}}$ is the random effect of the sire, $V_{D_{ijk}}$ the random effect of the dam nested within sire, $V_{C_{ijkl}}$ the random effect of container nested within sire and dam, and $V_{E_{ijklm}}$ is the environmental variation (i.e., residual error). Container was included as a random effect for hatch length and yolk sac volume to account for variation among replicates of each family. These replicate containers were split at the swim‐up stage for sampling/further rearing, which resulted in a single cup being used for swim‐up and juvenile measurements (i.e., cup not included in models for these traits). For the family‐level growth traits, the dam and container effects could not be estimated, and we controlled for among‐family variation by including sire as a random effect in the linear mixed models. The significance of the fixed effects in the linear mixed models was assessed using a Wald test implemented in the car package (Fox & Weisberg, 2011). We dropped the interaction term from the egg size models if it was found to be nonsignificant. For models with a significant population effect, traits were compared between populations using pairwise post hoc comparisons in the R package lsmeans (Lenth, 2016). We did not run a model for each trait with temperature as a fixed effect because the presence of population × temperature and egg size × temperature interactions prevented us from isolating the variance explained by population and egg size. Therefore, we ran the models separately at each temperature and then compared the results across the temperature treatments.

We estimated the variation explained by the individual fixed effects, the random effects, and the full model for the linear mixed models before and after including egg size as a covariate using the approach described by Nakagawa and Schielzeth (2013). Briefly, the fixed‐effect variation was calculated for the population and egg size effects separately by multiplying the design matrix of a given fixed effect by the vector of estimates for the same fixed effect. Random‐effect variation was quantified as the sum of the variation explained by all the random effects in the model (i.e., sire, dam, and cup variation). The variation accounted for by the full model was calculated as the sum of the fixed‐ and random‐effect variation. We presented the variation explained by the fixed effects, random effects, and full model as a proportion of the total variation, which was equal to the sum of the fixed‐effect, random‐effect, and residual variation. We excluded any traits that had a population x egg size interaction or did not have a relationship with egg size because the variance of population and egg size could not be separated or estimated. All the linear mixed models were fit using the lme4 package (Bates, Mächler, Bolker, & Walker, 2015).

### 2.8 Egg size and genetic architecture

For individual‐level traits, we also used a model comparison approach to quantify the influence of egg size on quantitative parameters (McAdam et al., 2014). Using the models described above, we compared the sire variance, dam variance, heritability, and maternal effects from linear mixed models before and after including egg size as a covariate for each trait. The heritability was calculated as four times *V*_*S*_ divided by the total phenotypic variance (*V*_*P*_ = *V*_*S*_ + *V*_*D*_ + *V*_*C*_ + *V*_*E*_) because *V*_*S*_ accounts for ¼ of the additive genetic variance when using a half‐sib, full‐sib breeding design (Falconer & Mackay, 1996). Maternal effects were calculated by subtracting *V*_*S*_ from *V*_*D*_, and then dividing by the total phenotypic variance (Lynch & Walsh, 1998). The linear mixed models were fit using the nlme package (Pinheiro, Bates, DebRoy, & Sarkar, 2016). The significance of the variance components was assessed using a simulation‐based restricted likelihood ratio test implemented using the RLRsim package in R (Scheipl, Greven, & Küchenhoff, 2008).

Bootstrap 95% confidence intervals (CIs) for the quantitative genetic parameters in the linear mixed models were estimated by resampling individuals within a family with replacement until the original sample size was replicated (Lynch & Walsh, 1998). Resampling of individuals was performed to account for within‐family variation, which allows for an unbiased calculation of the total phenotypic variance and prevents the overestimation of genetic effects (Puurtinen, Ketola, & Kotiaho, 2009). The resampled dataset was then used to estimate the variance components as well as the maternal effect and heritability. The resampling procedure was repeated for 5,000 iterations. Bias‐corrected and accelerated bootstrap confidence intervals were then calculated for each variance component, the maternal effect, and the heritability (Efron, 1987). The confidence intervals were used to determine whether there was a significant change in the parameters before and after including egg size as a covariate by assessing the overlap in the confidence intervals (i.e., no overlap = significant difference).

### 2.9 Egg size and divergence rates

The pairwise phenotypic divergence rates for egg size and the early life history traits between the populations were calculated using Haldanes (Gingerich, 1993). The Haldane is calculated as:

$$h\;=\;\frac{\left(\frac{\bar{X_{2}}}{S_{p}}\right)-\left(\frac{\bar{X_{1}}}{S_{p}}\right)}{g}$$

where $\bar{x_{1}}$ and $\bar{x_{2}}$ are the mean trait values for population 1 and population 2, *S*_*p*_ is the trait pooled standard deviation for the two populations, and *g* is the number of generations the populations have been separated (Gingerich, 1993; Hendry & Kinnison, 1999). Within each temperature treatment, the Haldanes were calculated for the early life history traits with and without controlling for egg size effects to show how much egg size differences contribute to the observed divergence rates. We used least squares means to control for egg size effects. Any early life history traits not correlated with egg size or that had an egg size x population interaction in the linear mixed models were excluded.

## 3 RESULTS

### 3.1 Egg size comparison among populations

The mean (mm ± *SE*) egg diameter of CR females (7.9 ± 0.10) was larger than that of both the PR (6.6 ± 0.09) and SR females (6.8 ± 0.09; *F*_2,65_ = 51.18, *p *<* *.001). The egg diameter of PR and SR females was no different. When female size was included as a covariate, egg diameter was positively related to female size (adj. *R* ^2^ = 0.70; *F*_1,63_ = 21.80, *p *<* *.001). After controlling for female size, the CR had a larger egg diameter than the PR, while the SR was no different than either population (*F*_2,63_ = 12.20, *p *<* *.001). The shift from an egg diameter difference between the CR and SR for the ANOVA to no difference for the ANCOVA indicates that the egg diameter difference between the populations is primarily driven by variation in female body size. In contrast, the difference in egg diameter between the CR and PR was maintained and was not explained by female body size.

### 3.2 Egg size and multivariate trait comparisons

**Figure 2:**
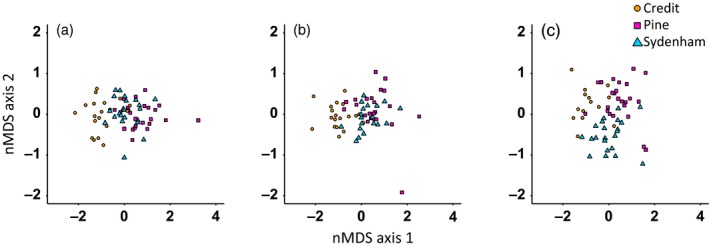
Nonmetric multidimensional scaling (nMDS) ordination plots of early life history traits from each population (Credit, Pine, and Sydenham rivers) when reared in a hatchery under (a) 6.5°C, (b) 9.4°C, and (c) 15.2°C temperature regimes. The trait matrices used for the ordination contained seven traits for the 6.5 and 9.4°C temperature treatments and five traits for the 15.2°C temperature treatment

Multivariate analysis revealed that the populations can be differentiated based on their early life history traits, with the strength of differentiation depending upon temperature regime (Figure 2, Table 1). The nMDS ordinations from each of the temperature treatments had stress values <15 and were considered acceptable representations of the data (6.5°C = 0.06; 9.4°C = 0.11; 15.2°C = 0.04). The nMDS ordination plots show that the clearest separation occurs between the CR and the Lake Huron populations, particularly at high temperature (Figure 2). Differentiation between the PR and SR was generally weak, but most apparent at 15.2°C (Figure 2). The PERMANOVAs confirmed the patterns of separation (Table 1). The population effect in the PERMANOVA was strongest for comparisons between the CR and the Lake Huron populations (*R* ^2^ values; Table 1). For the PR and SR comparisons, the population effect was weak at the 6.5 and 9.4°C and increased at 15.2°C.

**Table 1:** PERMANOVA results for the pairwise population comparisons of early life history trait matrices at each temperature treatment. The PERMANOVAs were run for 999 iterations

Temperature (°C)	Pairwise comparison	*F*‐statistic	*R* ^2^	*p*‐Value
6.5	Credit–Pine	*F*_1,42_ = 44.1	0.51	.001
	Credit–Sydenham	*F*_1,40_ = 20.3	0.34	.001
	Pine–Sydenham	*F*_1,44_ = 7.4	0.14	.002
9.4	Credit–Pine	*F*_1,41_ = 30.8	0.43	.001
	Credit–Sydenham	*F*_1,37_ = 23.9	0.39	.001
	Pine–Sydenham	*F*_1,40_ = 4.0	0.09	.01
15.2	Credit–Pine	*F*_1,36_ = 23.9	0.40	.001
	Credit–Sydenham	*F*_1,34_ = 19.7	0.37	.001
	Pine–Sydenham	*F*_1,44_ = 16.1	0.27	.001

The trait distance matrices were positively correlated with the egg diameter distance matrices for all temperature treatments (Mantel test; 6.5°C: *r*_M_ = 0.76, *p *=* *.001; 9.4°C: *r*_M_ = 0.70, *p *=* *.001; 15.2°C: *r*_M_ = 0.57, *p *=* *.001). This positive correlation indicates that large family‐wise differences in egg diameter are associated with large family‐wise differences in early life history traits. The correlation between the trait and egg size distance matrices weakened with increasing temperature.

### 3.3 Egg size and among‐population variation

All the individual early life history traits were different among the populations before controlling for egg size (Table 2). Offspring from the CR tended to be larger and grow faster than those from the PR or SR, regardless of the temperature treatment. The main exception was the yolk sac conversion efficiency of the PR offspring, which was generally higher than that of the CR and SR. Egg diameter was positively related to most of the early life history traits; however, yolk sac conversion efficiency was negatively related to egg diameter and there was no relationship between egg diameter and swim‐up to juvenile growth. When egg diameter was included in the analyses, the pattern of population differences depended on the temperature treatment (Table 2). In the 6.5°C treatment, there were slight changes in the population differences before and after controlling for egg size, but the CR and SR offspring generally performed better than the PR offspring. There were no clear patterns of population differences at 9.4°C (Table 2). Population differences at 15.2°C shifted after the hatching stage when controlling for egg size, and the PR offspring started to outperform the CR and SR offspring (Table 2). There were also several egg diameter x population interactions present, whereby the slopes of the egg diameter–trait relationships varied among populations (Table 2).

**Table 2:** Comparisons of yolk sac volume (mm^3^; YSV), hatch length (mm; HL), swim‐up length (mm; SL), juvenile length (mm; JL), yolk sac conversion efficiency (mm/mm^3^; YSCE), hatch to swim‐up growth (mm/∆D; HSGR), and swim‐up to juvenile growth (mm/∆D; SJGR) among progeny from the Credit River (C), Pine River (P), and Sydenham River (S) when reared under three different temperature treatments. The differences among the populations are presented as inequalities before (Base) and after (Egg) controlling for egg diameter variation in the analyses. If there was an egg diameter x population interaction, a comparison of slopes was provided (Pop. × Egg). The direction of the relationship between egg diameter and the traits is denoted with a superscript + or ‐. Only the population comparison is provided if there was no relationship between egg size and a trait

Temp. (°C)	Trait	Base	Egg	
		Pop.	Pop.	Pop. × Egg
6.5	YSV	C > S > P	[C = S] > P^+^	
	HL	C > [S = P]	C = S = P^+^	
	SL	C > S > P	S > [C = P]^+^	
	JL	C > S > P	C = [S > P]^+^	
	YSCE	P > S > C		[P > C] = S^−^
	HSGR	C > S > P	C = S = P^+^	
	SJGR	[C > P] = S		
9.4	YSV	C > [S = P]	[C > P] = S^+^	
	HL	C > [S = P]		P > [C = S]^+^
	SL	C > [S = P]	C = S = P^+^	
	JL	C > S > P	C = S = P^+^	
	YSCE	[P = S] > C		[S = P] > C^−^
	HSGR	[C > S] = P	C = [P > S]^+^	
	SJGR	[C = S] > P		
15.2	YSV	C > S > P		C = [S > P]^+^
	HL	C > [S = P]	C = S = P^+^	
	SL	C > P > S	P > [C = S]^+^	
	YSCE	P > [C = S]	[C = P] > S^−^	
	HSGR	[C = P] > S		P > [C = S]^+^

**Figure 3:**
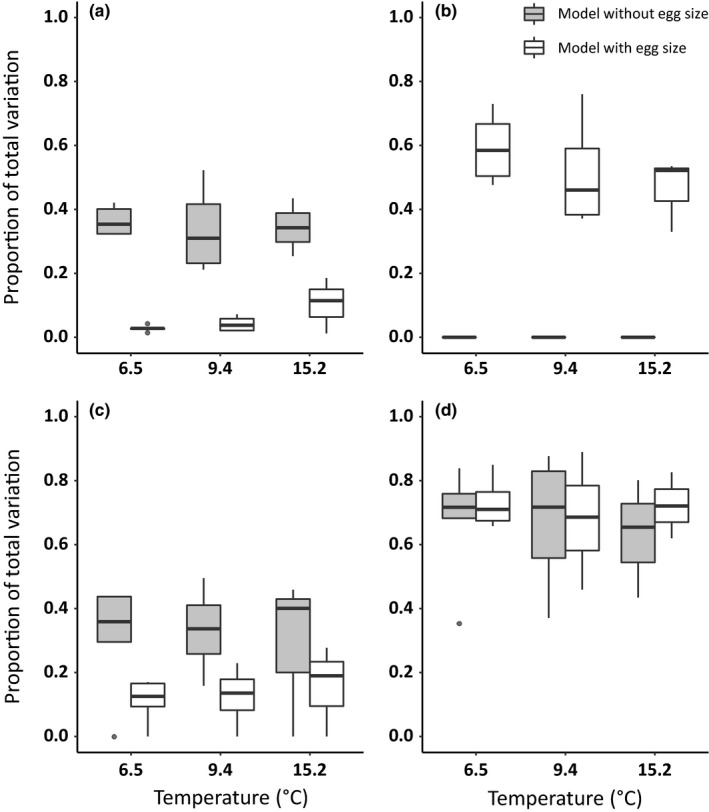
The proportion of variance explained by (a) the population fixed effect, (b) the egg size covariate, (c) the random effects, and (d) the fixed and random effects (i.e., full model) from linear mixed models comparing early life history traits among Chinook salmon populations at three temperatures. The linear mixed models were first fit with population as a fixed effect (gray) and then with population and egg size as fixed effects (white). Linear mixed models fit to length and volume data had sire and dam as random effects, whereas the growth traits had only sire as a random effect. Traits with a population x egg size interaction were excluded from this analysis because the individual effects of population and egg size could not be separated. The number of traits from 6.5, 9.4, and 15.2°C was 5, 4, and 3, respectively

Egg diameter explained a large proportion of the variation in the early life history traits, which affected the variance explained by the population fixed effect and random effects in the linear mixed models (Figure 3). The strongest effect of egg size was on the population effect (i.e., among‐population variation), which showed a large decline in the variance explained between the models before and after egg size was included as a covariate (Figure 3a). However, the relative change in the variance explained by the population effect was most pronounced at 6.5°C (mean ± *SE*: 95% ± 1.3) and decreased with increased temperature (9.4°C: 85% ± 6.5; 15.2°C: 73% ± 11.0). The variance explained by the random effects also decreased when egg size was included in the models, but to a lesser extent than the population effect (Figure 3c). Unlike the population effect, temperature did not appear to significantly influence the relative change in the random effect variance (6.5°C: 50% ± 13; 9.4°C: 66% ± 14; 15.2°C: 30% ± 17). The variance explained by the full models did not change before and after egg diameter was included in the models, indicating that the variation explained by egg size was accounted for by the population fixed effect and random effects in the models without egg diameter.

### 3.4 Egg size and genetic architecture

The dam variance was significant for most traits before and after controlling for egg diameter, and this was consistent across all temperature treatments, with the only exception being yolk sac volume at 15.2°C after controlling for egg diameter (Table 3). The addition of egg diameter into the models significantly reduced the dam variance components for all traits, regardless of temperature treatment (Table 3). The relative reduction of the dam variance components was consistently >60% (Table 3). In contrast, the sire variance was significant for only a few of the traits before and after controlling for egg diameter in the models, and the sire variance components were minimally influenced by the inclusion of egg size as a covariate (Table 3). The only traits to show a significant change in the sire variance were hatch length and yolk sac volume at 9.4°C.

**Table 3:** The estimated sire variance and dam variance for hatch length (HL), yolk sac volume (YSV), swim‐up length (SL), and juvenile length (JL) at each temperature treatment. The variance components were estimated using a linear mixed model without egg diameter as a covariate (Before) and with egg diameter included as a covariate (After). Values in the brackets are the bias‐corrected and accelerated bootstrap 95% confidence intervals. Bolded variance estimates are those with a significant difference between the estimates before and after the inclusion of egg diameter as a covariate in the models (i.e., nonoverlapping confidence intervals). The significance of the sire and dam variance components in the models was tested using a simulation‐based restricted likelihood ratio test (*p* < .05 = *)

Trait	Temp. (°C)	Sire variance		Dam variance	
		Before	After	Before	After
HL	6.5	0.02 (0–0.05)	0.01 (0.0–0.03)	0.46 (0.42–0.51)*	0.11 (0.08–0.14)*
	9.4	0.13 (0.10–0.16)*	0.04 (0.03–0.06)*	0.28 (0.24–0.33)*	0.05 (0.03–0.07)*
	15.2	0.02 (0–0.07)	0.004 (0.0–0.05)	0.40 (0.33–0.47)*	0.21 (0.15–0.27)*
YSV	6.5	66.6 (0–167.4)	0.0004 (0.0004–28.1)	994.0 (873.0–1124.1)*	171.3 (99.8–243.6)*
	9.4	562.8 (485.3–644.5)*	**142.9 (93.8–196.9)**	542.9 (462.6–626.7)*	132.3 (68.9–196.1)*
	15.2	0.0003 (0.0–0.92)	23.5 (0.0–62.8)	446.2 (390.8–503.7)*	**80.5 (33.7–129.5)**
SL	6.5	0.08 (0.02–0.13)	0.05 (0.02–0.09)	1.08 (0.98–1.17)*	0.21 (0.17–0.24)*
	9.4	0.11 (0.07–0.15)	0.05 (0.03–0.08)	0.98 (0.89–1.07)*	0.22 (0.17–0.24)*
	15.2	0.19 (0.14–0.24)	0.21 (0.16–0.26)*	0.71 (0.62–0.79)*	0.22 (0.16–0.25)*
JL	6.5	0.20 (0.03–0.36)	0.36 (0.22–0.50)*	2.33 (2.01–2.52)*	0.82 (0.60–0.91)*
	9.4	0.70 (0.52–0.88)*	0.67 (0.53–0.81)*	2.01 (1.65–2.26)*	0.96 (0.63–1.23)*

**Figure 4:**
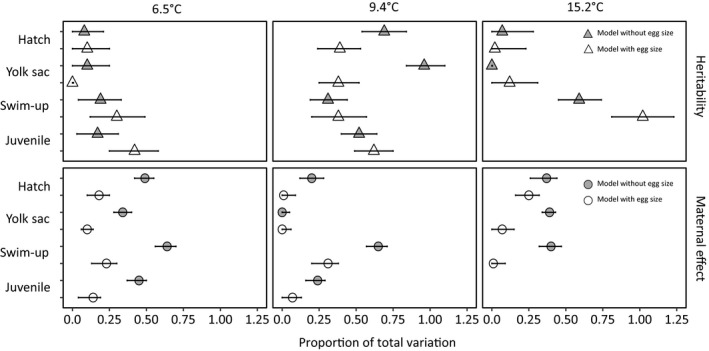
The heritability and maternal effect of hatch length, yolk sac volume, swim‐up length, and juvenile length at each temperature treatment. The quantitative genetic parameters were estimated before (gray) and after (white) including egg size as a covariate in the linear mixed models. Error bars are the bias‐corrected and accelerated bootstrap 95% confidence intervals

Maternal effects for the traits were influenced by the inclusion of egg diameter into the models, whereas the heritability was mostly unaffected. There was a significant reduction in the maternal effect for almost all traits at each temperature treatment when egg size was included into the models (nonoverlapping confidence intervals; Figure 4). Hatch length at 15.2°C and yolk sac volume at 9.4°C were the only traits to show no change in the maternal effect. The heritability was generally unchanged before and after controlling for egg size, except for a decrease in the heritability of hatch length and yolk sac volume at 9.4°C and an increase in the heritability of swim‐up length in 15.2°C (Figure 4).

The estimation of heritability and maternal effects was also influenced by temperature treatment. At 6.5°C, quantitative genetic analyses of hatch length, yolk sac volume, swim‐up length, and juvenile length showed that these traits were primarily influenced by maternal effects, which were significantly larger than the heritability (nonoverlapping confidence intervals for estimates from models without egg size; Figure 4). Maternal effects were also greater than genetic effects for swim‐up length and yolk sac conversion efficiency at 9.4 and 15.2°C, respectively (Figure 4). At 9.4°C, the heritability was high and maternal effects were low for hatch length, yolk sac volume, and juvenile length (Figure 4). At 15.2°C, maternal effects and heritability were no different for hatch length and swim‐up length (Figure 4).

### 3.5 Egg size and divergence rates

**Figure 5:**
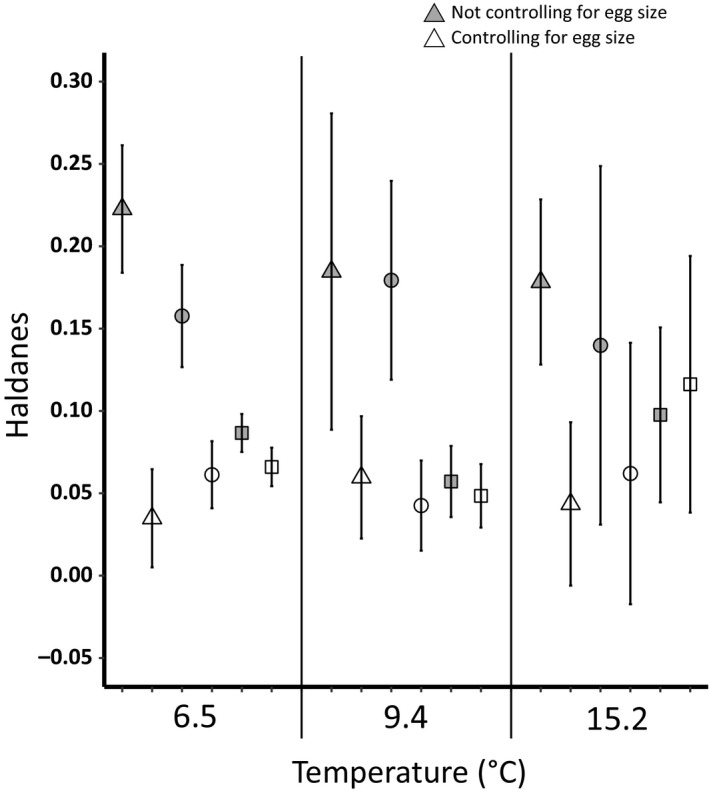
The mean (95% CI) divergence rate, in Haldanes, for the early life history traits before (gray) and after (white) controlling for egg size at each temperature. The divergence rates between the Credit–Pine (triangle), Credit–Sydenham (circle), and Sydenham–Pine (square) are presented separately. The mean and confidence intervals for the divergence rates were calculated using the estimated divergence rates of all traits within a temperature treatment for each pairwise population comparison. The number of traits used from 6.5, 9.4, and 15.2°C was 5, 4, and 3, respectively. The effect of egg size was controlled for using least squares means. Nonoverlapping confidence intervals indicate a significant difference between the Haldanes before and after controlling for egg size

The estimated pairwise divergence rates of the early life history traits were relatively high for divergence rates between the CR and the Lake Huron populations (PR and SR) before controlling for egg size, whereas the divergence rates were lower between the PR and SR (Figure 5). After controlling for egg diameter, there was a significant reduction in the estimated divergence rates at 6.5°C for the CR–PR and CR–SR comparisons, at 9.4°C for the CR–SR comparisons, and 15.2°C for the CR–PR comparisons (Figure 5). There were no significant differences between the PR–SR comparisons before and after controlling for egg size, regardless of temperature (Figure 5). The divergence rates for egg size were highest for the pairwise comparisons between the CR and the Lake Huron populations (CR–PR = 0.28 and CR–SR = 0.24), whereas the divergence rate between the PR and SR was relatively low (0.06).

## 4 DISCUSSION

Using a common‐garden hatchery study, we have shown that the early life history traits of introduced Great Lakes Chinook salmon populations have diverged within ~10 generations and that much of this divergence can be explained by variation in egg size. There was a strong relationship between egg size and most of the early life history traits, which resulted in egg size accounting for most of the among‐ and within‐population variation. Interestingly, the among‐population variation explained by egg size decreased with an increase in temperature. Although egg size explained much of the variation in the traits, population differences remained after controlling for egg size, suggesting that other effects, such as genetic, also contributed to the observed population differences. In general, our results are consistent with previous studies that have found evidence of population divergence among introduced salmonid populations on contemporary timescales (Haugen & Vøllestad, 2000; Hendry et al., 1998; Jensen et al., 2008; Kinnison et al., 1998; Koskinen et al., 2002; Thomassen, Barson, Haugen, & Vøllestad, 2011; Unwin, Quinn, Kinnison, & Boustead, 2000).

### 4.1 Egg size and among‐population variation

Egg size explained much of the among‐population variation in early life history traits across all the temperature treatments, which suggests that egg size variation is the primary driver of trait divergence among populations. Previous studies of introduced salmonids have mostly been interested in identifying the genetic effects underlying phenotypic divergence among populations (Hendry et al., 1998; Jensen et al., 2008; Kinnison et al., 1998; Koskinen et al., 2002; Thomassen et al., 2011; Unwin et al., 2000). However, our results suggest that any genetic effects contributing to the phenotypic divergence of early life history traits are minimal and that egg size can explain up to 100% of the among‐population variation. This has significant implications for the contemporary evolution of early life history traits because it suggests that egg size has a much greater capacity to alter offspring phenotypes in response to environmental changes than genetic effects on ecological timescales.

Phenotypic divergence rates are often used to quantify the among‐population variation in a phenotype per unit time (reviewed Hendry & Kinnison, 1999; Kinnison & Hendry, 2001). Prior to controlling for egg size, the phenotypic divergence rates of the early life history traits were similar to other studies of introduced salmonids, which have found divergence rates ranging from 0.007 to 0.36 (Haugen & Vøllestad, 2001; Hendry & Kinnison, 1999). These phenotypic divergence rates incorporate all components of a phenotype, such as genetic, maternal, and environmental effects (Hendry & Kinnison, 1999). Researchers often attempt to estimate the genetic divergences rates of traits (i.e., divergence attributable only to genetic effects; Reznick, Shaw, Rodd, & Shaw, 1997); however, few studies have attempted to assess the maternal contribution to divergence rates. Badyaev (2005) showed that degree of divergence among juvenile house finch (*Carpodacus mexicanus*) traits was positively related to the proportion of maternal variation underlying the traits. Similarly, we found that controlling for egg size reduced the mean divergence rate of the early life history traits for several of the pairwise population comparisons, indicating that egg size makes a significant contribution to population divergence.

### 4.2 Egg size and genetic architecture

Egg size was a strong maternal effect trait and significantly reduced both the dam variance and maternal effect for most of the early life history traits. Both the univariate and multivariate analyses revealed that there is strong relationship between the early life history traits and egg size across all temperatures regimes. This strong relationship between early life history traits and egg size is consistent with previous studies on salmonids (Beacham & Murray, 1985, 1990; Einum & Fleming, 2004; Haugen & Vøllestad, 2000; Hendry et al., 1998) and reflects the dependence of offspring development on the maternal per‐offspring allocation of resources (i.e., egg size and energy; Einum, Kinnison, & Hendry, 2004; Rollinson & Rowe, 2016). Although egg size explained most of the dam variance and maternal effect in our study, there are other potential sources of maternal variation, such as hormones, nutrients, immune factors, and mRNA (Brooks, Tyler, & Sumpter, 1997). Future studies can incorporate these additional maternal effect traits into models to better understand their relative importance to the expression of phenotypic variation in early life.

Maternal effects were generally stronger than additive genetic effects for the early life history traits, which has often been found by other quantitative genetic studies of salmonids (Falica et al., 2016; Heath et al., 1999; Houde et al., 2013; Kinnison et al., 1998; Páez et al., 2010; Pitcher & Neff, 2007). Because genetic effects are often weak during early life, one might conclude that early life history traits will have a limited capacity to adapt to new or changing environments (e.g., climate change), but this is at odds with the growing body of evidence from studies of contemporary evolution in salmonids (e.g., Haugen & Vøllestad, 2000; Hendry et al., 1998; Kinnison et al., 1998). The disconnect lies in the assumption that egg size variation, the primary maternal effect trait, is purely an environmental effect. Egg size is a heritable trait (Carlson & Seamons, 2008; Heath, Heath, Bryden, Johnson, & Fox, 2003; Kinnison, Unwin, Hendry, & Quinn, 2001), and the genes controlling egg size represent an indirect genetic effect that can influence the evolution of early life history traits (McAdam et al., 2014; Wolf, Brodie, Cheverud, Moore, & Wade, 1998). Therefore, changes in egg size could be an important pathway through which early life history traits could evolve in salmon even when the additive genetic effects are weak.

As the offspring progressed through ontogeny, there was no consistent reduction in the maternal effect with age. Previous studies have found that the maternal effect decreases as development progresses because offspring become more self‐reliant and additive genetic effects become more apparent (Heath et al., 1999; Wilson, Kruuk, & Coltman, 2005). Our experiment was terminated midway through the juvenile free‐feeding stage and likely did not provide enough time for the influence of maternal effects to disappear as in other longer term studies. Selection during the early life history stage is often size‐dependent (reviewed by Sogard, 1997), and the strong influence that maternal effects have on size‐related early life history traits indicates that aspects of the maternal environment, such as egg size, have important fitness consequences during this early developmental stage.

### 4.3 Egg size effect and temperature

The strength of the egg size effect on the among‐population variation in early life history traits decreased with an increase in rearing temperature. This suggests that the importance of egg size for mediating adaptation, in the context of introductions or climate change, might be lessened at elevated stream temperatures. Such a temperature‐dependent association was supported by the reduced effect of egg size on the among‐population variation at warm temperatures as well as the consistent decrease in the correlation between trait and egg size distance matrices with increased temperature. The temperature dependence of the egg size effect may be related to changes in the body size–metabolic rate relationship. Chinook salmon are adapted to cold‐water environments, and temperatures above ~12°C are considered to be stressful during early life, which is well below the highest temperature treatment used in our study (reviewed by Richter & Kolmes, 2005). Régnier et al. (2013) studied the relationship between metabolic rate and body size for brown trout (*Salmo trutta*) alevins across three temperature treatments and found that metabolic rate was highly variable and no longer scaled with body size at high temperature (14.5°C). As the body size of salmon progeny is positively related to egg size, a breakdown in the body size–metabolic rate relationship at high temperature would also impact the body size–egg size relationship and result in a reduced egg size effect on growth‐related early life history traits at high temperature. The standard error of hatch to swim‐up growth rate for salmon in our study did increase in the 15.2°C treatment for two populations, lending some support to the idea that changes in metabolic rate contributed to the weakened egg size effects at high temperature (Table S2).

An increase in the expression of additive genetic effects with temperature could be another explanation for the reduced effect of egg size on the among‐population variation at warm temperatures (Charmantier & Garant, 2005; Hoffmann & Merilä, 1999). Rearing in a stressful or novel environment can lead to the release of “cryptic” genetic variation, which increases the expression of additive genetic variance and reduces the maternal and/or environmental variance of a trait (Hayden, Ferrada, & Wagner, 2011; Lynch & Walsh, 1998; McGuigan, Nishimura, Currey, Hurwit, & Cresko, 2011; Purchase & Moreau, 2012; Rutherford, 2000). However, there was no consistent increase in the additive genetic variance or heritability of traits in the warm treatment relative to the colder treatments, which makes cryptic genetic variation an unlikely explanation for the reduced effect of egg size on the among‐population variation at warm temperatures.

Interestingly, the heritability was greater than maternal effects for hatch length, yolk sac volume, and juvenile length in the medium treatment, but not at any other temperature treatment (i.e., genotype x environment interaction). The change in the heritability with temperature was primarily due to an increase in the additive genetic variance at 9.4°C, and not due to a consistent change in the dam or environmental variance (Table S3). The increase in the heritability of the traits in the medium treatment is consistent with the results of a meta‐analysis by Charmantier and Garant (2005) who found that heritability was greater in “favorable” environmental conditions. We consider our medium treatment as the most favorable thermal condition because it is closest to the optimal growth temperature for Chinook salmon during their early life history stage (Richter & Kolmes, 2005). In contrast, the warm treatment may have been stressful enough to constrain the expression of additive genetic variation leading to the low additive genetic variance we observed for traits in that treatment. An implication of this finding is that the presence of environmental heterogeneity in the wild, such as differences in environmental conditions among salmon redds, could lead to spatial/temporal variation of genetic effects within a population making it difficult to predict the response of a population to selection (Charmantier & Garant, 2005). The change in the additive genetic variance with temperature also highlights the need for studies to rear populations under a range of possible environmental conditions likely to be experienced in the wild (if the wild is not logistically feasible) in order to more rigorously assess whether the evolution of phenotypic traits is constrained by a lack of additive genetic variation.

### 4.4 Egg size differences among populations

Variation in egg size among the Great Lakes Chinook salmon populations could be related to differences in maternal size and/or egg size selection regimes. There is a well‐known positive relationship between maternal size and egg size in salmonids (Einum et al., 2004), and the difference in egg size between the CR and SR was primarily explained by variation in maternal size‐at‐maturity. These differences in size‐at‐maturity are likely due to differences in growth opportunities within their respective lake environments because the SR and CR have a similar age‐at‐maturity (Haring et al., 2016; Suk et al., 2012). In contrast, maternal size could not explain the differences in egg size between the CR and PR. These populations continued to have an egg size difference even after controlling for female size, which might indicate that egg size is being differentially selected for in these populations. Egg size can be influenced by pre‐ and postzygotic selection pressures, such as variation in the temperature experienced by the mother during egg maturation (Jonsson & Jonsson, 2016), the natal stream temperature (Braun, Patterson, & Reynolds, 2013), the length of upstream migration (Kinnison et al., 2001), and the incubation gravel size (Quinn, Hendry, & Wetzel, 1995). Kinnison et al. (2001) found that salmon populations with a longer upstream migration produced smaller eggs than those with a shorter migration. The PR population has a much longer migration distance (>100 km) than the CR (~14 km) and, consistent with Kinnison et al. (2001), the PR produced smaller eggs. Further research is needed to disentangle the various environmental factors contributing to the egg size differences among the populations.

## 5 CONCLUSION

We provide evidence that the early life history traits of Great Lakes Chinook salmon populations have diverged within ~10 generations and that egg size explained most of the observed among‐population variation. However, the contribution of egg size to the among‐population variation decreased with an increase in temperature, indicating that other effects contribute at high temperature. Within populations, the dam variance and maternal effect were generally the most influential source of phenotypic variation, regardless of temperature. Egg size explained much of the maternal effect, suggesting that egg size is the primary maternal effect trait influencing offspring phenotypes. Overall, egg size appeared to mediate the primary response of early life history phenotypes when introduced into a new environment, while genetic effects provided a limited amount of additional phenotypic variation. These results highlight the integral role egg size plays in the contemporary evolution of fish early life history traits, and future studies are needed to better understand the genetic and environmental effects shaping egg size and offspring traits. Such studies will be required if we are to reliably predict the response of early life history traits to environmental change.

## DATA ARCHIVING STATEMENT

All phenotypic data used in this study are archived in the Dryad Digital Repository: https://doi.org/10.5061/dryad.r4ps0.
