# Supplementary material for: Egg size and the adaptive capacity of early life history traits in Chinook salmon (Oncorhynchus tshawytscha)
Source: Evol Appl. 2017 Sep 14;11(2):205–19. doi: 10.1111/eva.12531 (PMC5775492; doi:10.1111/eva.12531)
Supplement: Supplementary file 1 [file EVA-11-205-s001.docx]

**Supporting Information**


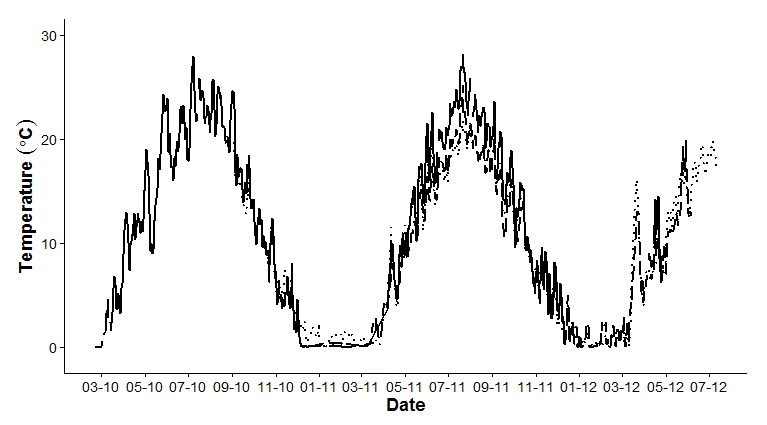


**Figure S1:** The temperature profiles from the Credit (solid), Pine (dash), and Sydenham (dotted) Rivers between 20 February 2010 and 11 July 2012. The dates are presented as month-year. Not all river systems have data for the entire duration of the graphed time period. The approximate spawning times for the three populations are indicated with vertical lines (solid = Pine River; dashed = Credit and Sydenham Rivers). The leftmost vertical lines represent the beginning of the spawning event for a given population and the rightmost represent the end of the spawning event. The Pine River and Sydenham River temperature data was collected using HOBO temperature loggers by M. Thorn and the Credit River temperature data was provided by the Credit Valley Conservation Authority.

**Table S1**: The number of families (# Fam.) and offspring (# Off.) used in the analysis yolk sac volume (YSV), hatch length (HL), swim-up length (SL), juvenile length (JL), yolk sac conversion efficiency (YSCE), hatch to swim-up growth (HSGR), and swim-up to juvenile growth (SJGR). The sample sizes are provided for each population and temperature treatment.

|  |  | **Credit River** | | **Pine River** | | **Sydenham River** | | **Total** | |
| --- | --- | --- | --- | --- | --- | --- | --- | --- | --- |
| **Temp.** | **Trait** | **# Fam.** | **# Off.** | **# Fam.** | **# Off.** | **# Fam.** | **# Off.** | **# Fam.** | **# Off.** |
| 6.5 °C | YSV | 20 | 898 | 25 | 1061 | 22 | 1151 | 67 | 3110 |
|  | HL | 20 | 917 | 25 | 1124 | 22 | 1187 | 67 | 3228 |
|  | SL | 20 | 644 | 25 | 839 | 22 | 779 | 67 | 2262 |
|  | JL | 20 | 628 | 26 | 843 | 22 | 792 | 68 | 2263 |
|  | YSCE* | 20 | 20 | 24 | 24 | 22 | 22 | 66 | 66 |
|  | HSGR* | 20 | 20 | 24 | 24 | 22 | 22 | 66 | 66 |
|  | SJGR* | 20 | 20 | 25 | 25 | 22 | 22 | 67 | 67 |
| 9.4 °C | YSV | 20 | 1119 | 23 | 1106 | 20 | 1147 | 63 | 3372 |
|  | HL | 20 | 1136 | 23 | 1168 | 20 | 1194 | 63 | 3498 |
|  | SL | 20 | 686 | 26 | 779 | 21 | 717 | 67 | 2182 |
|  | JL | 20 | 641 | 26 | 834 | 22 | 799 | 68 | 2274 |
|  | YSCE* | 20 | 20 | 23 | 23 | 19 | 19 | 62 | 62 |
|  | HSGR* | 20 | 20 | 23 | 23 | 19 | 19 | 62 | 62 |
|  | SJGR* | 20 | 20 | 26 | 26 | 21 | 21 | 67 | 67 |
| 15.2 °C | YSV | 14 | 500 | 24 | 418 | 22 | 810 | 60 | 1728 |
|  | HL | 14 | 627 | 24 | 808 | 22 | 984 | 60 | 2419 |
|  | SL | 20 | 640 | 26 | 800 | 22 | 733 | 68 | 2173 |
|  | YSCE* | 14 | 14 | 24 | 24 | 22 | 22 | 60 | 60 |
|  | HSGR* | 14 | 14 | 24 | 24 | 22 | 22 | 60 | 60 |

*Measurements of the growth traits were derived from family means. Therefore, the number of families used is equal to the number of offspring.

**Table S2**: Yolk sac volume (mm^3^; YSV), hatch length (mm; HL), swim-up length (mm; SL), juvenile length (mm; JL), yolk sac conversion efficiency (mm/mm^3^; YSCE), hatch to swim-up growth (mm/∆D; HSGR), and swim-up to juvenile growth (mm/∆D; SJGR) of progeny from the Credit River, Pine River, and Sydenham River when reared under three different thermal regimes. Data are presented as mean ± standard error. YSCE, HSGR, and SJGR were multiplied by 100 for presentation purposes.

| **Temp.** | **Trait** | **Credit** | **Pine** | **Sydenham** |
| --- | --- | --- | --- | --- |
| 6.5 °C | YSV | 223.7 (10.1) | 115.4 (5.7) | 146.7 (7.0) |
|  | HL | 22.8 (0.2) | 21.2 (0.2) | 21.7 (0.1) |
|  | SL | 32.5 (0.3) | 30.0 (0.2) | 30.8 (0.2) |
|  | JL | 38.1 (0.3) | 34.3 (0.4) | 36.0 (0.3) |
|  | YSCE | 4.5 (0.2) | 8.1 (0. 5) | 6.5 (0. 3) |
|  | HSGR | 3.4 (0.05) | 3.1 (0.04) | 3.3 (0.04) |
|  | SJGR | 2.1 (0.08) | 1.7 (0.07) | 1.9 (0.08) |
| 9.4 °C | YSV | 227.6 (9.5) | 114.5 (6.7) | 129.8 (7.1) |
|  | HL | 22.2 (0.1) | 21.1 (0.2) | 21.3 (0.2) |
|  | SL | 32.8 (0.2) | 30.7 (0.2) | 31.0 (0.2) |
|  | JL | 37.6 (0.3) | 34.2 (0.4) | 35.7 (0.3) |
|  | YSCE | 4.8 (0. 2) | 9.3 (0. 6) | 7.8 (0. 5) |
|  | HSGR | 3.4 (0.05) | 3.3 (0.03) | 3.2 (0.03) |
|  | SJGR | 1.7 (0.08) | 1.2 (0. 1) | 1.6 (0.08) |
| 15.2 °C | YSV | 129.7 (6.6) | 82.9 (4.0) | 106.7 (6.0) |
|  | HL | 20.4 (0.2) | 18.8 (0.2) | 19.3 (0.1) |
|  | SL | 29.5 (0.2) | 28.2 (0.2) | 27.4 (0.2) |
|  | YSCE | 7.4 (0. 4) | 11.7 (0. 5) | 8.1 (0. 5) |
|  | HSGR | 3.6 (0.07) | 3.6 (0.07) | 3.1 (0.04) |

**Table S3:** The sire variance (Vs), dam variance (Vd), cup variance (Vc), environmental variance (Ve), heritability (h^2^), and maternal effects (m) for hatch length (HL), yolk sac volume (YSV), swim-up length (SL), and juvenile length (JL) at each temperature treatment. The cup variance is present only for the hatching traits because family pairs were split at the swim-up stage for sampling/further rearing. The heritability was calculated as the proportion of phenotypic variance explained by additive genetic effects (4*(Vs/Vs+Vd+Vc+Ve)). The maternal effects were calculated as the proportion of phenotypic variance explained by dam identity minus the variance explained by sire identity ((Vd-V_S_)/Vs+Vd+Vc+Ve). The quantitative genetic components were calculated using a model without egg size as a covariate (Base) and with egg size included as a covariate (Egg). Values in the brackets are the bias corrected and accelerated bootstrap 95% confidence intervals. The significance of the sire and dam random effects in the models were tested using a simulation-based restricted likelihood ratio test (P < 0.05 = *; P ≥ 0.05 = ^ns^).

| **Trait** | **Model** | **Temp.** | **Vs** | **Vd** | **Vc** | **Ve** | **h^2^** | **m** |
| --- | --- | --- | --- | --- | --- | --- | --- | --- |
| HL | Base | 6.5 °C | 0.02 (0 - 0.05) | **0.46 (0.42 - 0.51)** | 0.10 (0.08 - 0.11) | 0.32 (0.31 - 0.36) | 0.08 (0 - 0.21) | 0.49 (0.42 - 0.55) |
|  |  | 9.4 °C | **0.13 (0.10 - 0.16)** | **0.28 (0.24 - 0.33)** | 0.07 (0.06 - 0.08) | 0.27 (0.26 - 0.29) | 0.69 (0.54 - 0.84) | 0.20 (0.12 - 0.28) |
|  |  | 15.2 °C | 0.02 (0 - 0.07) | **0.40 (0.33 - 0.47)** | 0.13 (0.11 - 0.15) | 0.48 (0.47 - 0.51) | 0.07 (0 - 0.28) | 0.37 (0.26 - 0.44) |
| YSV |  | 6.5 °C | 66.6 (0 - 167.4) | **994.0 (873.0 - 1124.1)** | 314.8 (211.9 - 344.3) | 1318.1 (1303 - 1415) | 0.10 (0 - 0.25) | 0.34 (0.28 - 0.40) |
|  |  | 9.4 °C | **562.8 (485.3 – 644.5)** | **542.9 (462.6 - 626.7)** | 319.0 (252.7 - 342.8) | 911.2 (897.2 - 970.3) | 0.96 (0.84 - 1.1) | 0.0 (0.0 - 0.05) |
|  |  | 15.2 °C | 0.0003 (0.0 – 0.92) | **446.2 (390.8 - 503.7)** | 161.8 (107.3 - 181.1) | 546.1 (534.7 - 597.6) | 0.000001 (0 - 0.004) | 0.39 (0.34 - 0.43) |
| SL |  | 6.5 °C | 0.08 (0.02 - 0.13) | **1.08 (0.98 - 1.17)** | - | 0.43 (0.36 - 0.59) | 0.19 (0.04 - 0.33) | 0.64 (0.56 - 0.70) |
|  |  | 9.4 °C | 0.11 (0.07 - 0.15) | **0.98 (0.89 - 1.07)** | - | 0.27 (0.26 - 0.30) | 0.31 (0.19 - 0.44) | 0.65 (0.57 - 0.71) |
|  |  | 15.2 °C | 0.19 (0.14 - 0.24) | **0.71 (0.62 - 0.79)** | - | 0.39 (0.37 - 0.46) | 0.59 (0.45 - 0.74) | 0.40 (0.32 - 0.47) |
| JL |  | 6.5 °C | 0.20 (0.03 - 0.36) | **2.33 (2.01 - 2.52)** | - | 2.25 (2.12 - 2.63) | 0.17 (0.03 - 0.31) | 0.45 (0.37 - 0.50) |
|  |  | 9.4 °C | **0.70 (0.52 - 0.88)** | **2.01 (1.65 - 2.26)** | - | 2.69 (2.56 - 2.99) | 0.52 (0.40 - 0.64) | 0.24 (0.16 - 0.29) |
| HL | Egg | 6.5 °C | 0.01 (0.0 – 0.03) | **0.11 (0.08 – 0.14)** | 0.10 (0.08 – 0.11) | 0.32 (0.31 – 0.36) | 0.10 (0.0 – 0.25) | 0.18 (0.10 – 0.25) |
|  |  | 9.4 °C | **0.04 (0.03 – 0.06)** | **0.05 (0.03 – 0.07)** | 0.07 (0.06 – 0.08) | 0.27 (0.26 – 0.29) | 0.39 (0.24 – 0.53) | 0.01 (-0.06 – 0.09) |
|  |  | 15.2 °C | 0.004 (0.0 – 0.05) | **0.21 (0.15 – 0.27)** | 0.14 (0.10 – 0.15) | 0.48 (0.47 – 0.51) | 0.02 (0.0 – 0.23) | 0.25 (0.16 – 0.32) |
| YSV |  | 6.5 °C | 0.0004  (0.0004 – 28.1) | **171.3 (99.8 – 243.6)** | 313.0 (219.7 – 340.7) | 1319.8 (1303 – 1406) | 0.0000009 (0.0 – 0.07) | 0.10 (0.06 – 0.14) |
|  |  | 9.4 °C | 142.9 (93.8 – 196.9) | **132.3 (68.9 – 196.1)** | 313.6 (251.5 – 336.0) | 911.4 (898.0 – 964.5) | 0.38 (0.25 – 0.52) | -0.007 (-0.08 – 0.06) |
|  |  | 15.2 °C | 23.5 (0.0 – 62.8) | 80.5 (33.7 – 129.5) | 162.2 (110.4 – 180.2) | 545.5 (532.3 – 601.2) | 0.12 (0.0 – 0.31) | 0.07 (-0.02 – 0.15) |
| SL |  | 6.5 °C | 0.05 (0.02 – 0.09) | **0.21 (0.17 – 0.24)** | - | 0.43 (0.36 – 0.59) | 0.30 (0.12 – 0.49) | 0.23 (0.13 – 0.30) |
|  |  | 9.4 °C | 0.05 (0.03 – 0.08) | **0.22 (0.17 – 0.24)** | - | 0.27 (0.26 – 0.30) | 0.38 (0.20 – 0.57) | 0.31 (0.20 – 0.38) |
|  |  | 15.2 °C | **0.21 (0.16 – 0.26)** | **0.22 (0.16 – 0.25)** | - | 0.39 (0.37 – 0.45) | 1.02 (0.81 – 1.23) | 0.01 (-0.09 – 0.09) |
| JL |  | 6.5 °C | **0.36 (0.22 – 0.50)** | **0.82 (0.60 – 0.91)** | - | 2.24 (2.12 – 2.63) | 0.42 (0.25 – 0.58) | 0.14 (0.04 – 0.19) |
|  |  | 9.4 °C | **0.67 (0.53 – 0.81)** | **0.96 (0.63 – 1.23)** | - | 2.69 (2.57 – 2.96) | 0.62 (0.49 – 0.75) | 0.07 (-0.02 – 0.13) |
